# Radiomic Analysis of Contrast-Enhanced Mammography With Different Image Types: Classification of Breast Lesions

**DOI:** 10.3389/fonc.2021.600546

**Published:** 2021-05-28

**Authors:** Simin Wang, Ning Mao, Shaofeng Duan, Qin Li, Ruimin Li, Tingting Jiang, Zhongyi Wang, Haizhu Xie, Yajia Gu

**Affiliations:** ^1^Department of Radiology, Fudan University Shanghai Cancer Center, Shanghai, China; ^2^Department of Oncology, Shanghai Medical College, Fudan University, Shanghai, China; ^3^Department of Radiology, Yantai Yuhuangding Hospital, Yantai, China; ^4^GE Healthcare, Shanghai, China

**Keywords:** contrast-enhanced mammography, radiomics, breast lesions, classification, high-energy, low-energy, dual-energy subtraction

## Abstract

**Objective:** A limited number of studies have focused on the radiomic analysis of contrast-enhanced mammography (CEM). We aimed to construct several radiomics-based models of CEM for classifying benign and malignant breast lesions.

**Materials and Methods:** The retrospective, double-center study included women who underwent CEM between November 2013 and February 2020. Radiomic analysis was performed using high-energy (HE), low-energy (LE), and dual-energy subtraction (DES) images from CEM. Datasets were randomly divided into the training and testing sets at a ratio of 7:3. The maximum relevance minimum redundancy (mRMR) method and least absolute shrinkage and selection operator (LASSO) logistic regression were used to select the radiomic features and construct the best classification models. The performances of the models were assessed by the area under the receiver operating characteristic curve (AUC) with a 95% confidence interval (CI). Leave-group-out cross-validation (LGOCV) for 100 rounds was performed to obtain the mean AUCs, which were compared by the Wilcoxon rank-sum test and the Kruskal–Wallis rank-sum test.

**Results:** A total of 192 women with 226 breast lesions (101 benign; 125 malignant) were enrolled. The median age was 48 years (range, 22–70 years). For the classification of breast lesions, the AUCs of the best models were 0.931 (95% CI: 0.873–0.989) for HE, 0.897 (95% CI: 0.807–0.981) for LE, 0.882 (95% CI: 0.825–0.987) for DES images and 0.960 (95% CI: 0.910–0.998) for all of the CEM images in the testing set. According to LGOCV, the models constructed with the HE images and all of the CEM images showed the highest mean AUCs for the training (0.931 and 0.938, respectively; *P* < 0.05 for both) and testing sets (0.892 and 0.889, respectively; *P* = 0.55 for both), which were significantly higher than those of the two models constructed with the LE and DES images in the training (0.912 and 0.899, respectively; all *P* < 0.05) and testing sets (0.866 and 0.862, respectively; all *P* < 0.05).

**Conclusions:** Radiomic analysis of CEM images was valuable for classifying benign and malignant breast lesions. The use of HE images or all three types of CEM images can achieve the best performance.

## Introduction

Among women, breast cancer is the most commonly diagnosed cancer worldwide and the leading cause of cancer death in 103 countries (1). In the face of such a disease burden, precise, and rapid diagnosis is crucial in clinical practice.

Mammography is commonly utilized for screening and diagnostic use with detection of breast lesions. However, its sensitivity can be as low as 30–50% in women with dense breasts (2–4). Breast magnetic resonance imaging (MRI) is a state-of-the-art technique with the highest sensitivity to detect breast cancer (5, 6). However, the false-positive findings (7–10), lengthy examination time, high cost, and lack of accessibility for all patients (11, 12) are limitations of MRI.

**Figure 1:**
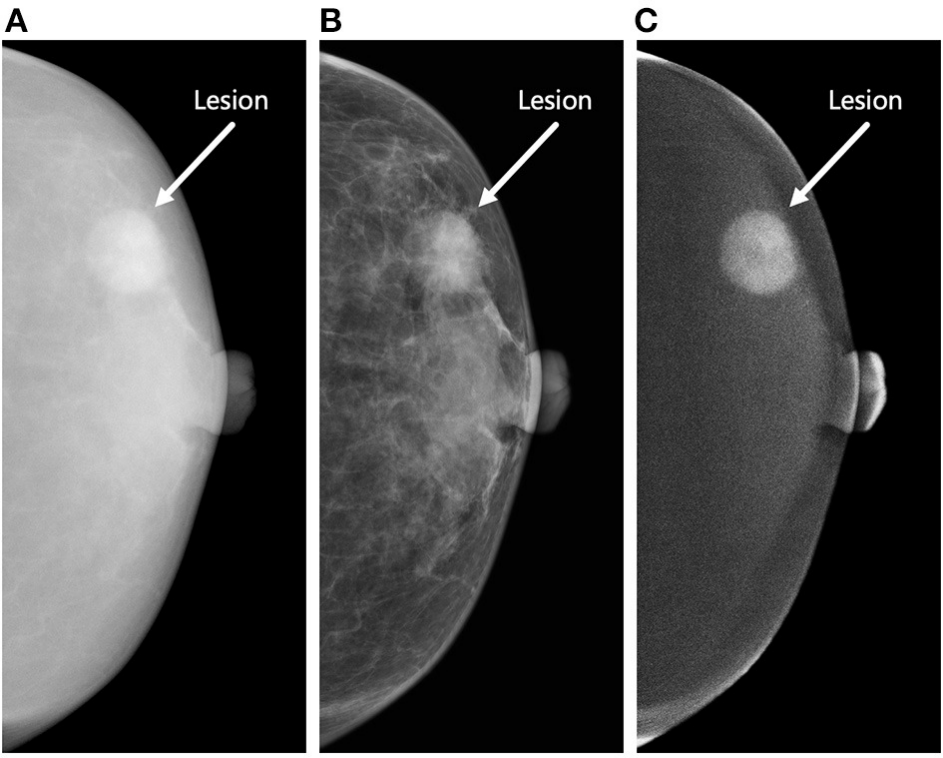
Three types of contrast-enhanced mammography (CEM) images of the craniocaudal view. The figure demonstrates a round-shaped lesion (white arrow) in the outer quadrant of the left breast of a 63-year-old woman. Biopsy revealed invasive ductal carcinoma (Grade II, Luminal A subtype). The lesion was correctly classified as malignant by the proposed radiomics model constructed by the combination of high-energy (HE), low-energy (LE), and dual-energy subtraction (DES) images. **(A)** The HE image of CEM. This type of image is not used for clinical diagnosis. **(B)** The LE image of CEM. This type of image is considered to be equivalent to conventional mammography. **(C)** The DES image of CEM. This type of image can highlight areas of contrast enhancement. The lesion shows marked enhancement in this image, whereas the patient shows minimal degree of background parenchymal enhancement.

Under such circumstances, contrast-enhanced mammography (CEM) has emerged (13). This methodology can demonstrate the morphological and angiogenic characteristics of breast lesions after the injection of iodine-based contrast material (14). CEM allows for obtaining three types of images for each craniocaudal (CC) and mediolateral oblique (MLO) view, including high-energy (HE), low-energy (LE), and dual-energy subtraction (DES) images (Figure 1). LE and DES images are used for clinical diagnosis, among which the former is considered to be equivalent to routine mammography (15, 16), and the latter can highlight areas of contrast enhancement (17).

In recent years, radiomics has been developing rapidly. It utilizes high-throughput computing to extract large numbers of image features and converts images into quantifiable data (18–20). Since CEM images can reflect both morphological and functional features of the lesions, such as MRI, and can have high spatial resolution comparable with that of mammography, we suspect that CEM would also have an encouraging application in the field of radiomics. Several studies have performed some preliminary work in this aspect (21–25), but the number of such studies is rather limited. Furthermore, it is worth noting that no studies thus far have used HE images in radiomic analysis since this type of image is not used for clinical diagnosis. Instead, they used LE images, DES images, or a combination of the two in their research. Although HE images are not used for clinical diagnosis (17), the subtle features of HE images may be mined with the help of radiomics. Therefore, we suppose that radiomic analysis of HE, LE, and DES images may contribute to the diagnosis of breast lesions. The purpose of this study is to construct radiomics-based models and to identify the model that can better classify breast lesions, which may be helpful for radiologists in decision-making.

## Materials and Methods

### Study Participants

This is a retrospective, double-center study. The Institutional Review Board and Ethics Committee of each center approved this study. The patient written informed consent was waived. We collected consecutive CEM images from the two institutions between November 2013 and February 2020. No study cohorts have been previously reported.

**Figure 2:**
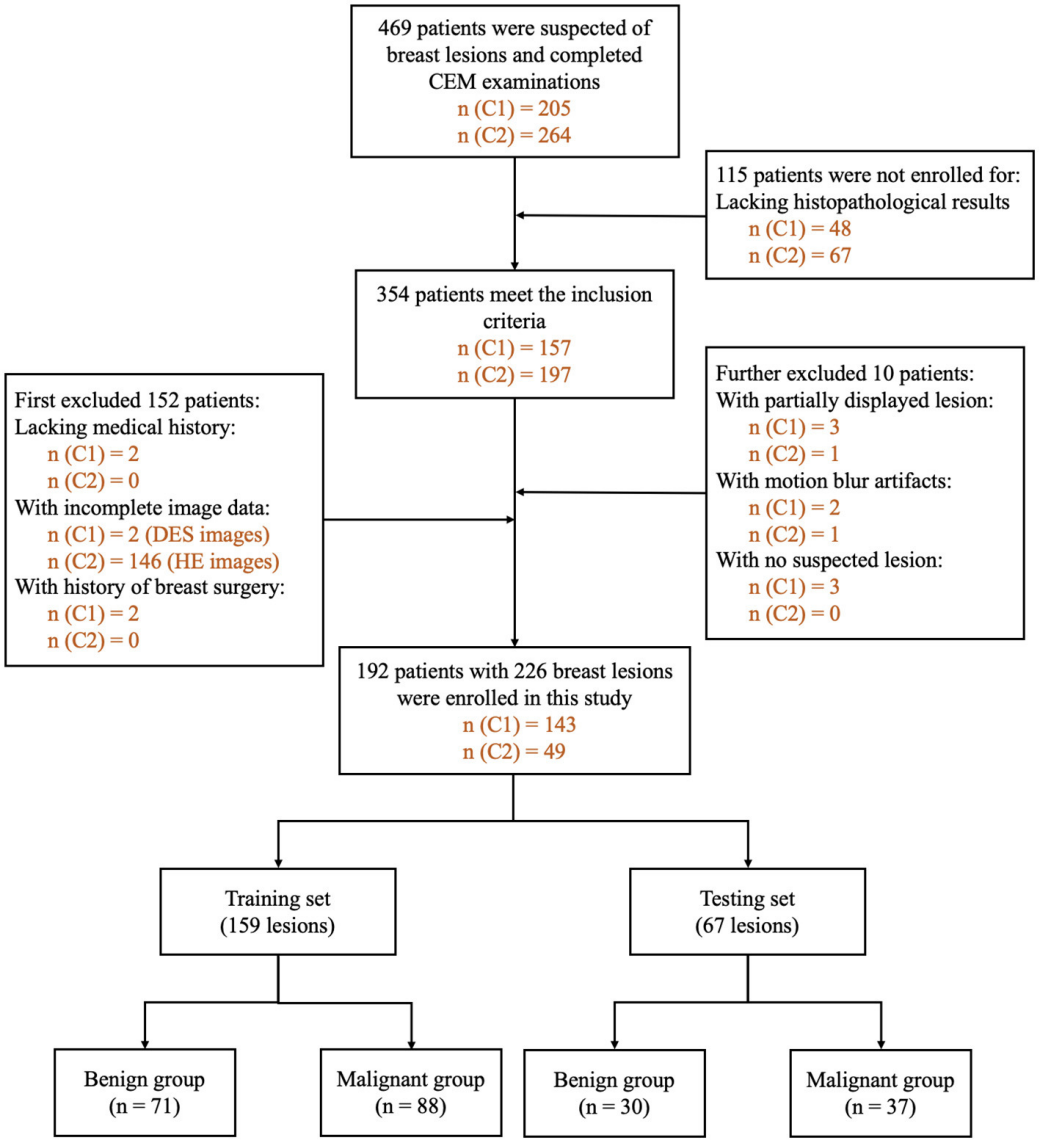
Flowchart of patient inclusion and exclusion criteria. n (C1): number of patients in Research Center 1. n (C2): number of patients in Research Center 2. DES, dual-energy subtraction; HE, high-energy; CEM, contrast-enhanced mammography.

The inclusion criteria were as follows: (1) patients with suspected breast lesions after physical examination or ultrasound and referred for CEM as part of diagnostic imaging, (2) patients who completed CEM examinations, and (3) patients with a final diagnosis that was confirmed by histopathology results within 2 weeks after CEM examination. We first excluded patients: (1) lacking medical history, (2) with missing or incomplete image data, and (3) with a history of breast surgery, breast radiotherapy, chemotherapy, or hormone treatment within 1 year prior to CEM examination. After preliminary evaluation of all the images, we further excluded patients (1) with images with poor image quality and (2) with no lesions detected on either HE, LE, or DES images. The flowchart of the patient inclusion and exclusion criteria is shown in Figure 2.

### CEM Image Interpretation by Human Readers

Two radiologists (NM and RL) with 8–10 years of experience in breast imaging and 5 years of experience interpreting CEM images reviewed the medical histories and the CEM images of all the potential participants and selected the eligible ones based on the inclusion and exclusion criteria. Several lesion characteristics were obtained by the radiologists according to the Breast Imaging Reporting and Data System (BI-RADS) lexicons for mammography and MRI (26). Breast density (a, b, c, or d) and lesion type (mass, calcification, architectural distortion, or asymmetry) characteristics were obtained from LE images. Degree of enhancement (no, mild, moderate, or marked enhancement), type of enhancement (focal, mass, or non-mass), and degree of background parenchymal enhancement (minimal, mild, moderate, or marked) characteristics were obtained from DES images.

### Reference Standard

The histopathological results obtained by biopsy or surgical specimens within 2 weeks after CEM examination are regarded as the reference standard for the classification of breast lesions in this study. None of the patients had undergone any form of treatment for the suspected lesions before the specimens were obtained. Lesions containing any invasive component or ductal carcinoma *in situ* are considered malignant; otherwise, the lesions are considered benign.

### CEM Examination

The examination protocols adopted by the two institutions were the same. All CEM examinations were performed using Senographe Essential mammography units (GE Healthcare, Buc, France). Before the examination, each patient rested for a while, and an intravenous catheter needle was placed in the antecubital fossa vein. A dose of 1.5 ml/kg body weight iodinated contrast material (iohexol, 300–350 mg I/ml; Beilu Pharmaceutical Co., Ltd., Beijing, China) was injected intravenously using an automated power injector at a flow rate of 3.0 ml/s, followed by a 10-ml bolus of saline. Two min after the injection of the contrast material, dual-energy exposures were performed using a fully automated exposure control method depending on the breast density and thickness. Bilateral CC views were obtained first, beginning with the suspicious breast. Then, bilateral MLO views were acquired in the same order. In a single projection, LE (peak tube voltage: 26–31 kVp) and HE (peak tube voltage: 45–49 kVp) exposures were performed continuously within 1.5 s to reduce motion artifacts. A proprietary algorithm was used to automatically reconstruct the HE and LE images to generate the DES images with the digital mammography unit. The total examination time of each patient since the injection of contrast material did not exceed 10 min. No contrast material-related adverse reactions were found in this study.

### Lesion Segmentation

For radiomic analysis, all of the CEM images were stored in the format of Digital Imaging and Communications in Medicine (DICOM) and loaded into an open-source image processing platform ITK-SNAP (version 3.6; www.itksnap.org) (27). Two radiologists (SW and QL) with 3–5 years of experience in breast imaging and 1 year of experience interpreting CEM images manually delineated the regions of interest (ROIs) together along the boundary of the lesions. Both of them were blinded to the patients' medical histories and histopathological results. A month later, they randomly selected 30 patients and resegmented the lesions to assess consistency for manual segmentation.

**Figure 3:**
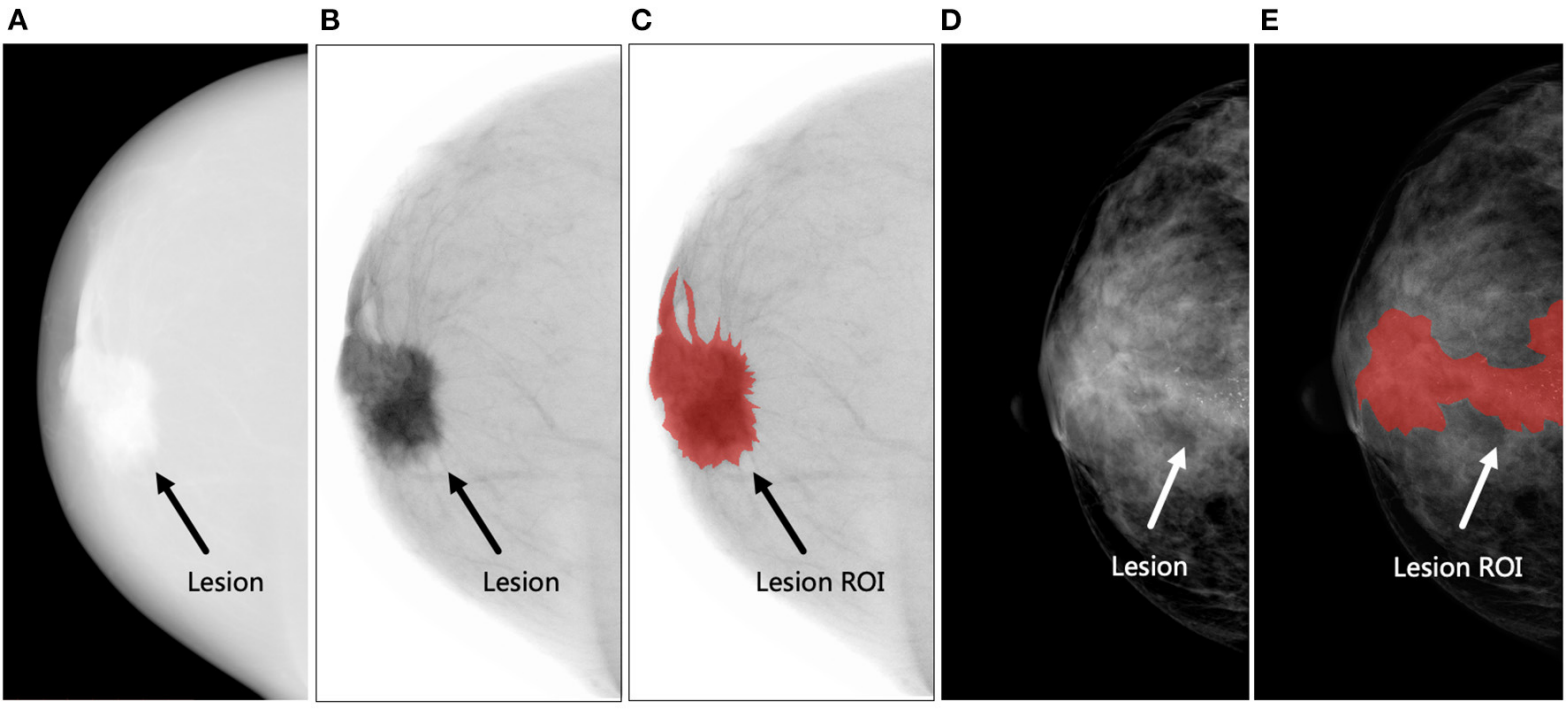
Examples of lesion segmentation. **(A–C)** The figures demonstrate an irregular-shaped lesion (black arrow) in the retro-areolar region of the right breast of a 60-year-old woman. Biopsy revealed invasive ductal carcinoma (Grade II, Luminal A subtype). The region of interest (ROI) was delineated on the negative film of the high-energy (HE) image. **(A)** The original HE image, **(B)** negative film of HE image, and **(C)** delineated lesion ROI (shown in red). **(D,E)** The figures demonstrate a lesion (white arrow) presented as microcalcifications without corresponding mass in the central area of the right breast of a 47-year-old woman. Biopsy revealed invasive ductal carcinoma with ductal carcinoma *in situ* (Grade III, human epidermal growth factor receptor-2-positive subtype). A closed loop was delineated along the edge of the microcalcifications as lesion ROI. **(D)** The low-energy image and **(E)** delineated lesion ROI (shown in red).

**Figure 4:**
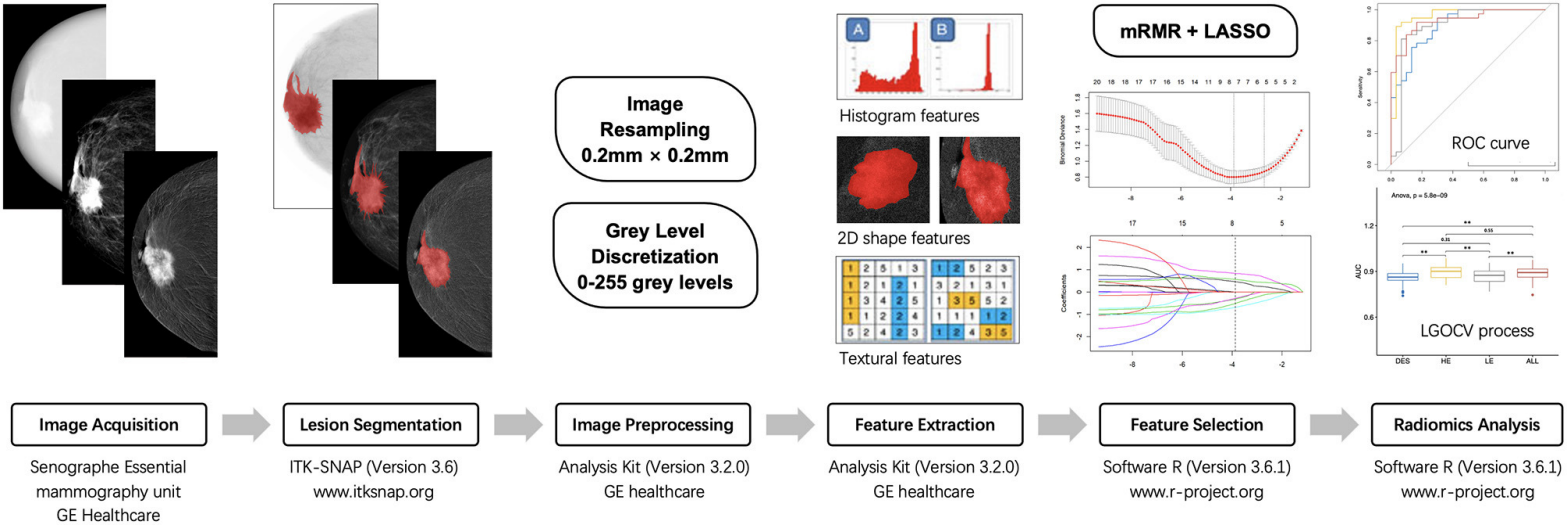
The workflow of radiomic analysis in the study. mRMR, maximum relevance minimum redundancy; LASSO, least absolute shrinkage and selection operator; ROC, receiver operating characteristic; LGOCV, leave-group-out cross-validation.

The criteria of lesion segmentation were as follows: (1) all HE images were transformed into negative films by the ITK-SNAP software for ROI delineation (Figures 3A–C); (2) for all patients, contours were separately delineated on HE, LE, and DES images of CC and MLO views if the lesions were visible on each image; if not, contours were delineated on either HE, LE, or DES images depending on which provided the preferable visualization of the lesion. Then, these contours were mapped onto other images, ensuring six ROIs for each lesion; (3) for lesions such as microcalcification, asymmetry, or architectural distortion without corresponding mass in LE images, closed loops were delineated along the edge of the lesions (Figures 3D,E); (4) multiple non-adjacent lesions were delineated separately and regarded as different lesions. The radiologists delineated all the suspicious lesions they had identified; (5) after lesion segmentation, another radiologist (TJ) who was not blinded to the histopathological results with 8 years of experience in breast imaging and 5 years of experience interpreting CEM images reviewed all the ROIs and deleted the ones without corresponding histopathological results. Therefore, only the pathologically proven lesions were retained in the following analysis.

### Feature Extraction

Before radiomic feature extraction, image preprocessing, including image resampling and gray level discretization, was performed. All voxel sizes of all images were resampled with the same size of 0.2 × 0.2 mm. Gray-level discretization was performed to discretize all the images to 256 gray levels. Then, the ROIs and the matched raw data were integrated into the Analysis Kit software (version 3.2.0; GE Healthcare) to extract the radiomic features. For each ROI, a feature dataset consisting of 392 radiomic features (including 42 histogram features, 5 shape features, and 345 textural features) was obtained (Supplementary Table 1). For each lesion, a total of six ROIs were delineated (HE-CC, HE-MLO, LE-CC, LE-MLO, DES-CC, and DES-MLO), thus producing six original feature datasets. All of the classification models used the radiomic features of both CC and MLO views in the following analysis.

### Feature Selection and Radiomics Model Construction

**Figure 5:**
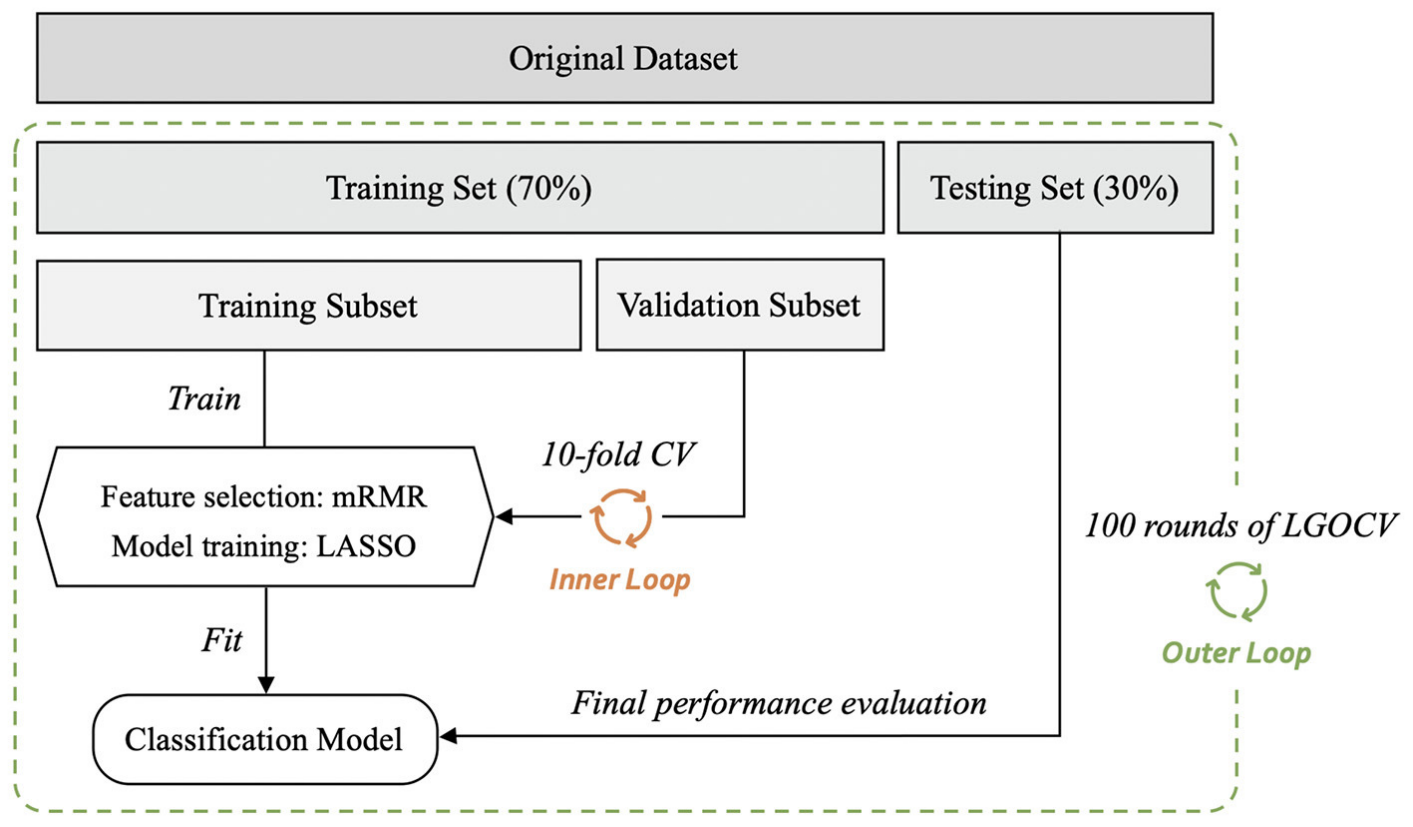
General structure of the radiomics-based classification model. After 100 rounds of leave-group-out cross-validation (LGOCV), the mean values of the area under the receiver operating characteristic curve, sensitivity, and specificity of the models were generated. mRMR, maximum relevance minimum redundancy; CV, cross-validation.

The radiomics classification model was used to differentiate malignant from benign lesions. The workflow of the study is shown in Figure 4, and the general structure of the radiomics model is shown in Figure 5.

Before constructing the models, each dataset was randomly divided into training and testing sets (training *vs*. testing set; 7:3) using the stratified random sampling method. The training set was further divided into the training and validation subsets to perform 10-fold cross-validation. For radiomic feature selection, we performed a three-step procedure. First, for the assessment of consistency for manual segmentation, the interobserver agreement test was conducted to choose the features that were not sensitive to the variation of manual segmentation. The features with interclass correlation coefficients <0.75 were kept in the datasets and used for constructing the classification models. Second, the maximum relevance minimum redundancy (mRMR) method (28) was performed to select the most relevant and least redundant radiomic features. Twenty features were retained for subsequent analyses. Third, least absolute shrinkage and selection operator (LASSO) logistic regression was used to find the most predictive subsets of features and to construct the corresponding classification models. As a sparse penalized aggression approach, LASSO regression (29, 30) has many desirable properties for regression models with a large number of covariates (31, 32) and can reduce variability and improve model accuracy. The penalty parameters of the models were tuned through 10-fold cross-validation, thus yielding the best classification model. The testing set was independently used to evaluate the performance of the established model without being involved in model construction or parameter tuning. The performances of the models were evaluated in terms of area under the receiver operating characteristic (ROC) curve (AUC) value with a 95% confidence interval (CI). The accuracy, sensitivity, and specificity of the models were also calculated by selecting an optimal threshold based on Youden's Index.

Considering the variation and sampling bias due to the random split of the datasets, we employed a nested cross-validation method by further conducting 100 rounds of leave-group-out cross-validation (LGOCV) to obtain 100 AUCs and sensitivity and specificity values, which are shown as means ± standard deviations. To compare the mean AUC values of different models, non-parametric tests were adopted, including the Wilcoxon rank-sum test for comparisons between two groups and the Kruskal–Wallis rank-sum test for analysis of variance.

### Statistical Analysis

All statistical analyses were performed using the statistical software R (version 3.6.1; www.r-project.org). The LASSO logistic regression and ROC curve analyses were conducted using the glmnet and the pROC software packages. Student's *t*-test was used to compare between the benign and malignant groups for the continuous variables, and Chi-square test or Fisher's Exact test was used for the categorical variables, as appropriate. The false discovery rate correction was also performed for multiple comparison. A two-sided *P*-value of less than 0.05 was considered statistically significant.

## Results

### Study Population and Lesion Characteristics

The patient and lesion characteristics are given in Table 1. A total of 192 patients with 226 pathologically proven lesions were included in our study. The median age of the patients was 48 years (range, 22–70 years). Of the 192 patients, 145 (75.5%) had dense breasts (c or d), and 65 (33.9%) had moderate to marked background parenchymal enhancement. Bilateral lesions were found in 20 patients, and multiple lesions were found in 10 patients.

**Table 1:** Basic patient and lesion characteristics.

**Characteristics**	**Training set (*****n*** **=** **159)**		***P*-value**	**Testing set (*****n*** **=** **67)**		***P*-value**	**Total (*****n*** **=** **226)**		***P*-value**
	**Benign (*n* = 71)**	**Malignant (*n* = 88)**		**Benign (*n* = 30)**	**Malignant (*n* = 37)**		**Benign (*n* = 101)**	**Malignant (*n* = 125)**	
Age (year)*	44.4 ± 9.7	51.2 ± 8.5	<0.001	40.2 ± 8.8	50.1 ± 10.5	<0.001	43.1 ± 9.6	50.9 ± 9.1	<0.001
Lesion size (mm)*	20.4 ± 14.6	29.8 ± 17.4	<0.001	17.7 ± 12.4	27.5 ± 12.3	0.001	19.6 ± 14.0	29.1 ± 16.1	<0.001
Breast density**			0.330			0.074			0.167
a	3/71 (4.2)	1/88 (1.1)		0/30 (0.0)	3/37 (8.1)		3/101 (3.0)	4/125 (3.2)	
b	14/71 (19.7)	25/88 (28.4)		2/30 (6.7)	8/37 (21.6)		16/101 (15.8)	33/125 (26.4)	
c	33/71 (46.5)	42/88 (47.7)		17/30 (56.7)	19/37 (51.4)		50/101 (49.5)	61/125 (48.8)	
d	21/71 (29.6)	20/88 (22.7)		11/30 (36.7)	7/37 (18.9)		32/101 (31.7)	27/125 (21.6)	
Mass**			0.212			0.227			0.066
Present	39/71 (54.9)	58/88 (65.9)		15/30 (50.0)	25/37 (67.6)		54/101 (53.5)	83/125 (66.4)	
Absent	32/71 (45.1)	30/88 (34.1)		15/30 (50.0)	12/37 (32.4)		47/101 (46.5)	42/125 (33.6)	
Microcalcification**			0.011			0.013			<0.001
Present	14/71 (19.7)	35/88 (39.8)		5/30 (16.7)	18/37 (48.6)		19/101 (18.8)	53/125 (42.4)	
Absent	57/71 (80.3)	53/88 (60.2)		25/30 (83.3)	19/37 (51.4)		82/101 (81.2)	72/125 (57.6)	
Architectural distortion**			0.005			0.068			<0.001
Present	1/71 (1.4)	14/88 (15.9)		1/30 (3.3)	8/37 (21.6)		2/101 (2.0)	22/125 (17.6)	
Absent	70/71 (98.6)	74/88 (84.1)		29/30 (96.7)	29/37 (78.4)		99/101 (98.0)	103/125 (82.4)	
Asymmetry **			1.000			0.067			0.409
Present	10/71 (14.1)	13/88 (14.8)		6/30 (20.0)	1/37 (2.7)		16/101 (15.8)	14/125 (11.2)	
Absent	61/71 (85.9)	75/88 (85.2)		24/30 (80.0)	36/37 (97.3)		85/101 (84.2)	111/125 (88.8)	
Degree of enhancement***			<0.001			0.002			<0.001
No enhancement	13/71 (18.3)	1/88 (1.1)		4/30 (13.3)	0/37 (0.0)		17/101 (16.8)	1/125 (0.8)	
Mild enhancement	34/71 (47.9)	20/88 (22.7)		16/30 (53.3)	9/37 (24.3)		50/101 (49.5)	29/125 (23.3)	
Moderate enhancement	8/71 (11.3)	24/88 (27.3)		2/30 (6.7)	11/37 (29.7)		10/101 (9.9)	35/125 (28.0)	
Marked enhancement	16/71 (22.5)	43/88 (48.9)		8/30 (26.7)	17/37 (45.9)		24/101 (23.8)	60/125 (48.0)	
Type of enhancement***			0.629			1.000			0.797
Focal enhancement	0/58 (0.0)	0/87 (0.0)		0/26 (0.0)	0/37 (0.0)		0/84 (0.0)	0/124 (0.0)	
Mass enhancement	39/58 (67.2)	63/87 (72.4)		17/26 (65.4)	23/37 (62.2)		56/84 (66.7)	86/124 (69.4)	
Non-mass enhancement	19/58 (32.8)	24/87 (27.6)		9/26 (34.6)	14/37 (37.8)		28/84 (33.3)	38/124 (30.6)	
Degree of BPE***			0.024			0.014			<0.001
Minimal	24/71 (33.8)	32/88 (36.4)		5/30 (16.7)	14/37 (37.8)		29/101 (28.7)	46/125 (36.8)	
Mild	17/71 (23.9)	34/88 (38.6)		6/30 (20.0)	14/37 (37.8)		23/101 (22.8)	48/125 (38.4)	
Moderate	13/71 (18.3)	15/88 (17.0)		11/30 (36.7)	6/37 (16.2)		24/101 (23.8)	21/125 (16.8)	
Marked	17/71 (23.9)	7/88 (8.0)		8/30 (26.7)	3/37 (8.1)		25/101 (24.8)	10/125 (8.0)	

* *Data are shown as means ± standard deviations. Other data are shown as proportions with percentages in parentheses*.

*Student's t-test was used to compare between the benign and malignant groups for age and lesion size. Chi-square test or Fisher's Exact test was used to test the differences for categorical variables, as appropriate. A P-value less than 0.05 is considered statistically significant*.

** *Lesion characteristics are obtained from low-energy (LE) images of contrast-enhanced mammography*.

*** *Lesion characteristics are obtained from dual-energy subtraction (DES) images of contrast-enhanced mammography*.

*BPE, background parenchymal enhancement*.

Of the 226 lesions, 101 (44.7%) were benign, including 41 (40.6%) fibroadenomas, 37 (36.6%) adenoses, 14 (13.9%) intraductal papillomas, 5 (5.0%) inflammations, and 4 (4.0%) other benign lesions; 125 (55.3%) were malignant, consisting of 107 (85.6%) invasive ductal carcinomas, 8 (6.4%) ductal carcinomas *in situ*, 4 (3.2%) invasive lobular carcinomas, 2 (1.6%) papillary carcinomas, 2 (1.6%) mucinous carcinomas, and 2 (1.6%) other malignant lesions.

### Performances of the Best Classification Models

**Figure 6:**
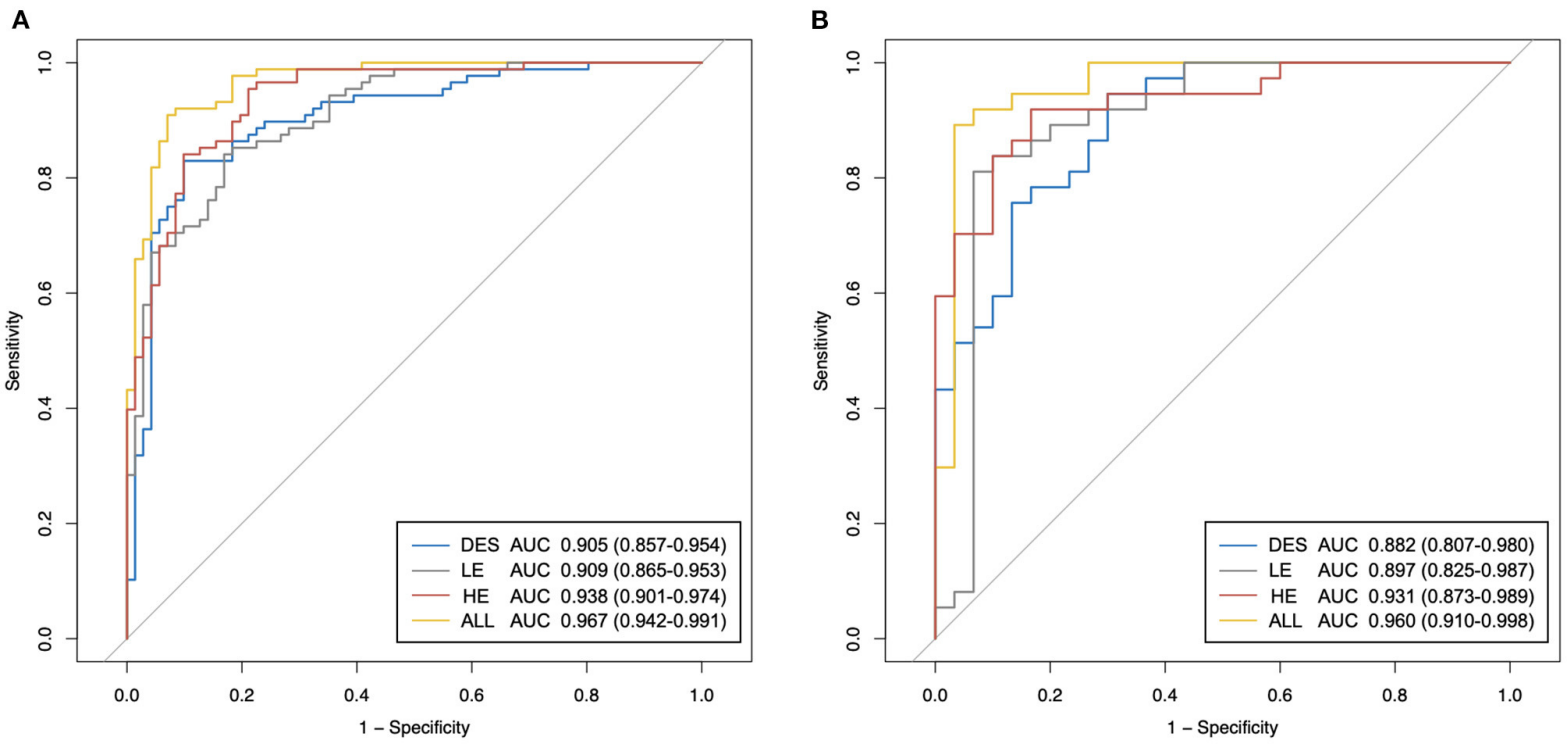
The receiver operating characteristic (ROC) curves of the proposed best classification models of different types of contrast-enhanced mammography (CEM) images. **(A)** ROC curves in the training set. **(B)** ROC curves in the testing set. HE, high-energy; LE, low-energy; DES, dual-energy subtraction; “ALL” stands for a combination of HE, LE, and DES images.

The performances of the best classification models of different types of CEM images are shown in Table 2 and Figure 6. The selected radiomic features and their corresponding coefficients are provided in Supplementary Figure 1.

**Table 2:** The performances of the best classification models.

**Models**	**Training set**				**Testing set**			
	**AUC (95% CI)**	Accuracy*	Sensitivity*	Specificity*	**AUC (95% CI)**	Accuracy*	Sensitivity*	Specificity*
Model 1: HE	0.938 (0.901–0.974)	88.1 (140/159)	95.5 (84/88)	78.9 (56/71)	0.931 (0.873–0.989)	89.6 (60/67)	91.9 (34/37)	83.3 (25/30)
Model 2: LE	0.909 (0.865–0.953)	85.5 (136/159)	81.8 (72/88)	90.1 (64/71)	0.897 (0.825–0.987)	85.1 (57/67)	81.1 (30/37)	90.0 (27/30)
Model 3: DES	0.905 (0.857–0.954)	84.9 (135/159)	84.1 (74/88)	85.9 (61/71)	0.882 (0.807–0.980)	83.6 (56/67)	83.8 (31/37)	83.3 (25/30)
Model 4: HE + LE + DES	0.967 (0.942–0.991)	91.8 (146/159)	90.9 (80/88)	93.0 (66/71)	0.960 (0.910–0.998)	89.6 (60/67)	91.9 (34/37)	86.7 (26/30)

*All of the models are constructed by the radiomic features of both craniocaudal (CC) and mediolateral oblique (MLO) views*.

* *Data are shown as percentages with proportions in parentheses*.

*HE, high-energy; LE, low-energy; DES, dual-energy subtraction; AUC, area under the receiver operating characteristic curve; CI, confidence interval*.

In both the training and testing sets, the performances of the models generated by any type of CEM images (Table 2: Models 1–3) were fairly good, with all AUCs <0.882 and all accuracy values <83.8%. Importantly, the model constructed by the combination of HE, LE, and DES images (Table 2: Model 4) yielded the best overall performance [AUC for the testing set: 0.960 (95% CI: 0.910–0.998); accuracy for the testing set: 89.7%]. In addition, Model 1 constructed by the HE images presented good overall diagnostic performance [AUC for the testing set: 0.931 (95% CI: 0.873–0.989); accuracy for the testing set: 88.2%] among the three types of CEM images, followed by the other two models with similar AUCs [AUC for Model 2 in the testing set: 0.897 (95% CI: 0.825–0.987); AUC for Model 3 in the testing set: 0.882 (95% CI: 0.807–0.980)].

In terms of sensitivity, in both the training and the testing sets, Model 1 and Model 4 still ranked first (sensitivity = 91.9% for both in the testing set), followed by Model 3 (sensitivity = 83.8% in the testing set). Model 2 constructed by LE images showed the lowest sensitivity in both the training and testing sets (sensitivity = 81.1% in the testing set). In terms of specificity in the testing set, Model 2 ranked first (specificity = 90.0%), followed by Model 4 (specificity = 86.7%), and both Model 1 and Model 3 had specificity values of 83.3%. All of the models showed similar trends in the training and testing sets in terms of AUC, accuracy, sensitivity, and specificity.

### LGOCV Analysis

After 100 rounds of LGOCV, the obtained mean values of the AUC, sensitivity, and specificity are displayed in Table 3.

**Table 3:** The results of leave-group-out cross-validation (LGOCV) analysis.

**Models**	**Training set**			**Testing set**		
	**AUC**	**Sensitivity**	**Specificity**	**AUC**	**Sensitivity**	**Specificity**
Model 1: HE	0.931 ± 0.021	0.895 ± 0.034	0.881 ± 0.042	0.892 ± 0.040	0.887 ± 0.075	0.818 ± 0.108
Model 2: LE	0.912 ± 0.020	0.798 ± 0.055	0.887 ± 0.054	0.866 ± 0.045	0.801 ± 0.110	0.850 ± 0.114
Model 3: DES	0.899 ± 0.022	0.838 ± 0.044	0.857 ± 0.076	0.862 ± 0.039	0.832 ± 0.059	0.824 ± 0.084
Model 4: HE + LE + DES	0.938 ± 0.013	0.903 ± 0.049	0.853 ± 0.047	0.889 ± 0.038	0.883 ± 0.083	0.838 ± 0.100

*Data are shown as means ± standard deviations*.

*HE, high-energy; LE, low-energy; DES, dual-energy subtraction; AUC, area under the receiver operating characteristic curve*.

**Figure 7:**
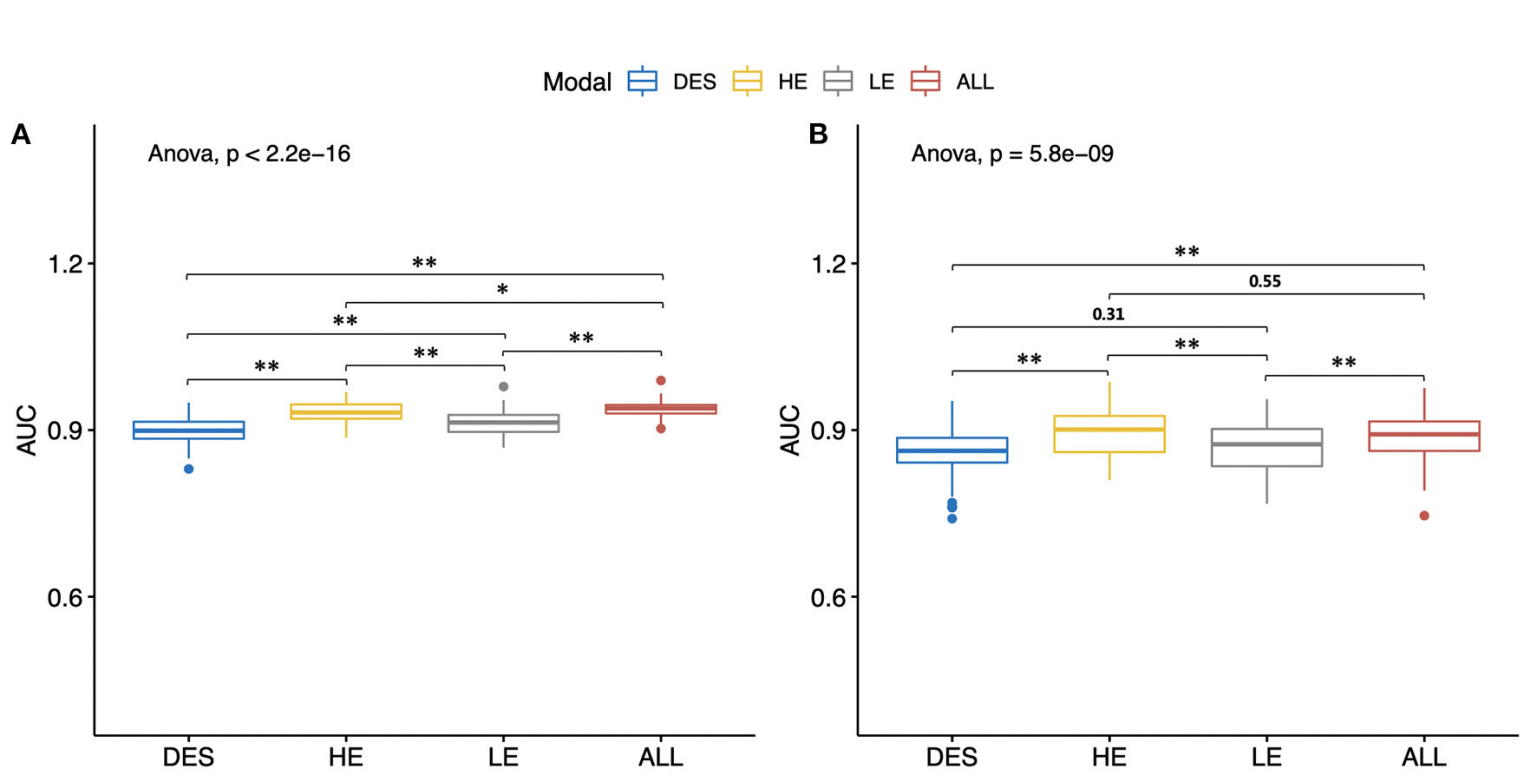
Mean values of the areas under the receiver operating characteristic curves (AUCs) of different types of contrast-enhanced mammography images. All of the models were constructed by the radiomic features of both craniocaudal and mediolateral oblique views. The Wilcoxon rank-sum test and the Kruskal–Wallis rank-sum test were used to compare the mean AUC values of different models. The false discovery rate correction was performed for multiple comparison. **(A)** Mean AUCs in the training set. **(B)** Mean AUCs in the testing set. HE, high-energy; LE, low-energy; DES, dual-energy subtraction; “ALL” stands for a combination of HE, LE, and DES images. *P < 0.05. **P < 0.01.

Consistent with the performances of the abovementioned best models, the mean AUCs generated by any type of CEM images (Table 3: Models 1–3) were still good, with all mean AUCs <0.862 in both the training and testing sets. In both the training and testing sets, the differences of mean AUCs of all the models (Table 3: Models 1–4) were statistically significant (Figure 7, both *P* < 0.01). In the testing set, Model 1 constructed by HE images and Model 4 constructed by the combination of HE, LE, and DES images still achieved the highest levels of AUCs after 100 rounds of LGOCV (mean AUC = 0.892 ± 0.040 for Model 1; mean AUC = 0.889 ± 0.038 for Model 4; *P* = 0.55), followed by the other two models (mean AUC = 0.866 ± 0.045 for Model 2; mean AUC = 0.862 ± 0.039 for Model 3; *P* = 0.31). The mean AUCs of Model 1 and Model 4 were significantly higher than those of Model 2 and Model 3 (all *P* < 0.01) in the testing set, which was consistent with the results of the best classification models without conducting LGOCV analysis (Table 2).

In the testing set, Model 1 and Model 4 still reached high levels of mean sensitivity (mean sensitivity = 88.7% for Model 1; mean sensitivity = 88.3%), followed by Model 3 (mean sensitivity = 83.2%) and Model 2 (mean sensitivity = 80.1%). In terms of specificity, all of the models have reached good mean specificity levels ranging from 85.9 to 88.7% in the training set and from 81.8 to 85.0% in the testing set. It is worth noting that the mean specificity value of Model 2 constructed by LE images still ranked first in both the training and testing sets (mean specificity = 88.7% for the training set; mean specificity = 85.0% for the testing set), which was in line with the results of the best classification models (Table 2: Model 2).

Furthermore, we combined the original six datasets to generate six other datasets for each lesion (Supplementary Figure 2). We also provided several heatmaps showing the median AUCs of all the models constructed with the 12 datasets to make the results more intuitive (Supplementary Figure 3). The median AUCs are essentially in parallel with the mean AUCs of the corresponding models.

## Discussion

Our study has proposed a feasible radiomic analysis method for CEM images for the differentiation of benign and malignant breast lesions. The findings in our study have shown that the models constructed with any type of CEM images show good performances, among which the model constructed with HE images performed the best. When the model employs the radiomic features of all three types of images, it can always achieve fairly satisfactory results with a high level of robustness. The result suggests that all CEM images contribute to the diagnosis of breast lesions to some extent, probably because they can reflect diverse image characteristics containing complementary information. Importantly, although HE images are currently thought to be clinically uninterpretable, they may contain useful information as original images without being postprocessed and may be valuable in the field of radiomics. Furthermore, we found that the radiomic analysis for DES images alone is not as ideal as those of the others. This finding is similar to another study (22) in which DES images were considered to have lost some heterogeneity information due to the digital subtraction process.

To the best of our knowledge, this is the first study to fully evaluate the diagnostic performances of all types of CEM images with use of radiomics. In addition, the numbers of benign and malignant cases are relatively balanced, which may reduce the potential classification bias toward the majority of cases and the consequent overfitting problem. Some previous studies have employed undersampling (21) or oversampling (22) techniques under these circumstances, but we attempted to avoid the problem from the origin.

In our study, we have defined the method of lesion segmentation in detail by converting HE images into negative images and mapping the optimal ROIs from one type of image to the other type. In practice, we found that after converting HE images into negative films and adjusting the window level and window width, the outline of the lesion became clearer and could be delineated effectively. Additionally, most of the patients in our research had dense breasts, which are common in Asian women; therefore, it was difficult to accurately segment the lesions in LE images in some cases. Thus, the method of mapping the optimal ROIs among different types of CEM images is advisable and sometimes even necessary in women with extremely dense breasts. One previous study concluded that by mapping the optimal lesion segmentation from DES images onto LE images, the classification performance can be significantly improved (22). Some studies used the radiomic features of either CC or MLO view images (21, 33), or both of them (23, 24), whereas another study (22) used the mean value of two feature values separately computed from CC and MLO view images to represent the final feature value. Instead, we used the radiomic features of both CC and MLO view images, hoping to make the best use of the image information.

In addition to the 10-fold cross-validation, we further conducted 100 rounds of LGOCV to validate the performances of the models. The AUCs of different models were relatively stable since they showed essentially the same trend before and after the LGOCV step. Since the results of radiomic analysis can be affected by the data to some extent, this cross-validation method can minimize the case partition bias.

Two meta-analyses reported the pooled specificity of CEM in the diagnosis of breast cancer to be 58–84% (34, 35), which denotes the discrepancies for specificity between studies and leaves room for further improvement in the diagnostic accuracy. The results of our study have shown the potential to improve the specificity, with the highest mean specificity value <84% (Table 3: Model 2). If our results are further substantiated in future prospective studies, the invasive biopsies of benign lesions may probably be reduced by the help of non-invasive radiomic analysis of CEM images. However, it seems that the HE and DES images do not contribute greatly to the improvement of the specificity obtained with the LE images, which still needs further exploration.

Our study had the following limitations. First, we mixed the data from two research centers to train and test the models rather than using the data from Center 2 for independent external testing; furthermore, all of the CEM examinations were performed on the same type of equipment. These factors limit the extrapolation of our conclusions. Second, since this is a retrospective study, some image data, especially HE images, were missing, which led to a smaller sample size than expected. Further prospective research with a larger sample size is warranted. Third, we manually delineated the contours of the lesions, which may affect the repeatability of the research. Fourth, the sensitivities in our study were not as high as the ones reported by human readers (34, 35), which may need further improvement in future studies. Finally, the dataset in our study was enriched for malignant lesions, thus likely overestimating the models' performances to some extent.

In summary, we proposed a radiomics-based method to classify benign and malignant breast lesions using CEM images and found that all of the HE, LE, and DES images of CEM can provide valuable information in the process, among which HE images seem to perform better than the others. It is recommended that all CEM images should be used in radiomic analysis to obtain the most satisfactory and stable performance in breast lesion classification.

## Data Availability Statement

The raw data supporting the conclusions of this article will be made available by the authors, without undue reservation.

## Ethics Statement

The studies involving human participants were reviewed and approved by the Ethics Committee of Shanghai Cancer Center and the Ethics Committee of Yantai Yuhuangding hospital. Written informed consent for participation was not required for this study in accordance with the national legislation and the institutional requirements.

## Author Contributions

SW: study design. SW and ZW: data collection. RL and NM: image evaluation. SW and NM: manuscript drafting. All authors listed have made a substantial, direct and intellectual contribution to the work, and approved it for publication.

## Conflict of Interest

SD is an employee of General Electric (GE) Healthcare (Shanghai, China). The remaining authors declare that the research was conducted in the absence of any commercial or financial relationships that could be construed as a potential conflict of interest.

## Abbreviations

AUC: area under the receiver operating characteristic curve
BI-RADS: breast imaging reporting and data system
CC: craniocaudal
CEM: contrast-enhanced mammography
CI: confidence interval
DES: dual-energy subtraction
DICOM: digital imaging and communications in medicine
HE: high-energy
LASSO: least absolute shrinkage and selection operator
LE: low-energy
LGOCV: leave-group-out cross-validation
MLO: mediolateral oblique
MRI: magnetic resonance imaging
mRMR: maximum relevance minimum redundancy
ROC: receiver operating characteristic
ROI: region of interest.
