# Supplementary material for: Radiomic Analysis of Contrast-Enhanced Mammography With Different Image Types: Classification of Breast Lesions
Source: Front Oncol. 2021 May 28;11:600546. doi: 10.3389/fonc.2021.600546 (PMC8195270; doi:10.3389/fonc.2021.600546)
Supplement: Supplementary file 1 [file Table_1.docx]

**Supplemental Table**

Supplemental Table 1. Radiomic features from different categories.

|  | First-order features | GLCM features | GLRLM features | GLSZM features | Shape features |
| --- | --- | --- | --- | --- | --- |
| 1 | Percentile5 | ClusterProminence_AllDirection_offset1 | HighGreyLevelRunEmphasis_AllDirection_offset1 | GreyLevelNonuniformity_AllDirection_offset1 | Elongation |
| 2 | Percentile10 | ClusterProminence_AllDirection_offset1_SD | HighGreyLevelRunEmphasis_AllDirection_offset1_SD | GreyLevelNonuniformity_AllDirection_offset1_SD | Major axis length |
| 3 | Percentile15 | ClusterProminence_AllDirection_offset4 | HighGreyLevelRunEmphasis_AllDirection_offset4 | GreyLevelNonuniformity_AllDirection_offset4 | Maximum 2D diameter |
| 4 | Percentile20 | ClusterProminence_AllDirection_offset4_SD | HighGreyLevelRunEmphasis_AllDirection_offset4_SD | GreyLevelNonuniformity_AllDirection_offset4_SD | Minor Axis length |
| 5 | Percentile25 | ClusterProminence_AllDirection_offset7 | HighGreyLevelRunEmphasis_AllDirection_offset7 | GreyLevelNonuniformity_AllDirection_offset7 | Pixel surface |
| 6 | Percentile30 | ClusterProminence_AllDirection_offset7_SD | HighGreyLevelRunEmphasis_AllDirection_offset7_SD | GreyLevelNonuniformity_AllDirection_offset7_SD |  |
| 7 | Percentile35 | ClusterProminence_angle0_offset1 | HighGreyLevelRunEmphasis_angle0_offset1 | GreyLevelNonuniformity_angle0_offset1 |  |
| 8 | Percentile40 | ClusterProminence_angle0_offset4 | HighGreyLevelRunEmphasis_angle0_offset4 | GreyLevelNonuniformity_angle0_offset4 |  |
| 9 | Percentile45 | ClusterProminence_angle0_offset7 | HighGreyLevelRunEmphasis_angle0_offset7 | GreyLevelNonuniformity_angle0_offset7 |  |
| 10 | Percentile50 | ClusterProminence_angle135_offset1 | HighGreyLevelRunEmphasis_angle135_offset1 | GreyLevelNonuniformity_angle135_offset1 |  |
| 11 | Percentile55 | ClusterProminence_angle135_offset4 | HighGreyLevelRunEmphasis_angle135_offset4 | GreyLevelNonuniformity_angle135_offset4 |  |
| 12 | Percentile60 | ClusterProminence_angle135_offset7 | HighGreyLevelRunEmphasis_angle135_offset7 | GreyLevelNonuniformity_angle135_offset7 |  |
| 13 | Percentile65 | ClusterProminence_angle45_offset1 | HighGreyLevelRunEmphasis_angle45_offset1 | GreyLevelNonuniformity_angle45_offset1 |  |
| 14 | Percentile70 | ClusterProminence_angle45_offset4 | HighGreyLevelRunEmphasis_angle45_offset4 | GreyLevelNonuniformity_angle45_offset4 |  |
| 15 | Percentile75 | ClusterProminence_angle45_offset7 | HighGreyLevelRunEmphasis_angle45_offset7 | GreyLevelNonuniformity_angle45_offset7 |  |
| 16 | Percentile80 | ClusterProminence_angle90_offset1 | HighGreyLevelRunEmphasis_angle90_offset1 | GreyLevelNonuniformity_angle90_offset1 |  |
| 17 | Percentile85 | ClusterProminence_angle90_offset4 | HighGreyLevelRunEmphasis_angle90_offset4 | GreyLevelNonuniformity_angle90_offset4 |  |
| 18 | Percentile90 | ClusterProminence_angle90_offset7 | HighGreyLevelRunEmphasis_angle90_offset7 | GreyLevelNonuniformity_angle90_offset7 |  |
| 19 | Percentile95 | ClusterShade_AllDirection_offset1 | LowGreyLevelRunEmphasis_AllDirection_offset1 | Size zone variability |  |
| 20 | Quantile0.025 | ClusterShade_AllDirection_offset1_SD | LowGreyLevelRunEmphasis_AllDirection_offset1_SD | High intensity emphasis |  |
| 21 | Quantile0.250 | ClusterShade_AllDirection_offset4 | LowGreyLevelRunEmphasis_AllDirection_offset4 | High intensity large area emphasis |  |
| 22 | Quantile0.500 | ClusterShade_AllDirection_offset4_SD | LowGreyLevelRunEmphasis_AllDirection_offset4_SD | High intensity small area emphasis |  |
| 23 | Quantile0.750 | ClusterShade_AllDirection_offset7 | LowGreyLevelRunEmphasis_AllDirection_offset7 | Low intensity emphasis |  |
| 24 | Quantile0.975 | ClusterShade_AllDirection_offset7_SD | LowGreyLevelRunEmphasis_AllDirection_offset7_SD | Low intensity large area emphasis |  |
| 25 | Energy | ClusterShade_angle0_offset1 | LowGreyLevelRunEmphasis_angle0_offset1 | Low intensity small area emphasis |  |
| 26 | Entropy | ClusterShade_angle0_offset4 | LowGreyLevelRunEmphasis_angle0_offset4 | Intensity variability |  |
| 27 | Frequency size | ClusterShade_angle0_offset7 | LowGreyLevelRunEmphasis_angle0_offset7 | Large area emphasis |  |
| 28 | Kurtosis | ClusterShade_angle135_offset1 | LowGreyLevelRunEmphasis_angle135_offset1 | Small area emphasis |  |
| 29 | Max intensity | ClusterShade_angle135_offset4 | LowGreyLevelRunEmphasis_angle135_offset4 | Zone percentage |  |
| 30 | Min intensity | ClusterShade_angle135_offset7 | LowGreyLevelRunEmphasis_angle135_offset7 |  |  |
| 31 | Mean deviation | ClusterShade_angle45_offset1 | LowGreyLevelRunEmphasis_angle45_offset1 |  |  |
| 32 | Mean value | ClusterShade_angle45_offset4 | LowGreyLevelRunEmphasis_angle45_offset4 |  |  |
| 33 | Median intensity | ClusterShade_angle45_offset7 | LowGreyLevelRunEmphasis_angle45_offset7 |  |  |
| 34 | Range | ClusterShade_angle90_offset1 | LowGreyLevelRunEmphasis_angle90_offset1 |  |  |
| 35 | Relative deviation | ClusterShade_angle90_offset4 | LowGreyLevelRunEmphasis_angle90_offset4 |  |  |
| 36 | Root mean square | ClusterShade_angle90_offset7 | LowGreyLevelRunEmphasis_angle90_offset7 |  |  |
| 37 | Skewness | Correlation_AllDirection_offset1 | LongRunEmphasis_AllDirection_offset1 |  |  |
| 38 | Standard deviation | Correlation_AllDirection_offset1_SD | LongRunEmphasis_AllDirection_offset1_SD |  |  |
| 39 | Uniformity | Correlation_AllDirection_offset4 | LongRunEmphasis_AllDirection_offset4 |  |  |
| 40 | Variance | Correlation_AllDirection_offset4_SD | LongRunEmphasis_AllDirection_offset4_SD |  |  |
| 41 | Volume count | Correlation_AllDirection_offset7 | LongRunEmphasis_AllDirection_offset7 |  |  |
| 42 | Voxel value sum | Correlation_AllDirection_offset7_SD | LongRunEmphasis_AllDirection_offset7_SD |  |  |
| 43 |  | Correlation_angle0_offset1 | LongRunEmphasis_angle0_offset1 |  |  |
| 44 |  | Correlation_angle0_offset4 | LongRunEmphasis_angle0_offset4 |  |  |
| 45 |  | Correlation_angle0_offset7 | LongRunEmphasis_angle0_offset7 |  |  |
| 46 |  | Correlation_angle135_offset1 | LongRunEmphasis_angle135_offset1 |  |  |
| 47 |  | Correlation_angle135_offset4 | LongRunEmphasis_angle135_offset4 |  |  |
| 48 |  | Correlation_angle135_offset7 | LongRunEmphasis_angle135_offset7 |  |  |
| 49 |  | Correlation_angle45_offset1 | LongRunEmphasis_angle45_offset1 |  |  |
| 50 |  | Correlation_angle45_offset4 | LongRunEmphasis_angle45_offset4 |  |  |
| 51 |  | Correlation_angle45_offset7 | LongRunEmphasis_angle45_offset7 |  |  |
| 52 |  | Correlation_angle90_offset1 | LongRunEmphasis_angle90_offset1 |  |  |
| 53 |  | Correlation_angle90_offset4 | LongRunEmphasis_angle90_offset4 |  |  |
| 54 |  | Correlation_angle90_offset7 | LongRunEmphasis_angle90_offset7 |  |  |
| 55 |  | GLCMEnergy_AllDirection_offset1 | ShortRunEmphasis_AllDirection_offset1 |  |  |
| 56 |  | GLCMEnergy_AllDirection_offset1_SD | ShortRunEmphasis_AllDirection_offset1_SD |  |  |
| 57 |  | GLCMEnergy_AllDirection_offset4 | ShortRunEmphasis_AllDirection_offset4 |  |  |
| 58 |  | GLCMEnergy_AllDirection_offset4_SD | ShortRunEmphasis_AllDirection_offset4_SD |  |  |
| 59 |  | GLCMEnergy_AllDirection_offset7 | ShortRunEmphasis_AllDirection_offset7 |  |  |
| 60 |  | GLCMEnergy_AllDirection_offset7_SD | ShortRunEmphasis_AllDirection_offset7_SD |  |  |
| 61 |  | GLCMEnergy_angle0_offset1 | ShortRunEmphasis_angle0_offset1 |  |  |
| 62 |  | GLCMEnergy_angle0_offset4 | ShortRunEmphasis_angle0_offset4 |  |  |
| 63 |  | GLCMEnergy_angle0_offset7 | ShortRunEmphasis_angle0_offset7 |  |  |
| 64 |  | GLCMEnergy_angle135_offset1 | ShortRunEmphasis_angle135_offset1 |  |  |
| 65 |  | GLCMEnergy_angle135_offset4 | ShortRunEmphasis_angle135_offset4 |  |  |
| 66 |  | GLCMEnergy_angle135_offset7 | ShortRunEmphasis_angle135_offset7 |  |  |
| 67 |  | GLCMEnergy_angle45_offset1 | ShortRunEmphasis_angle45_offset1 |  |  |
| 68 |  | GLCMEnergy_angle45_offset4 | ShortRunEmphasis_angle45_offset4 |  |  |
| 69 |  | GLCMEnergy_angle45_offset7 | ShortRunEmphasis_angle45_offset7 |  |  |
| 70 |  | GLCMEnergy_angle90_offset1 | ShortRunEmphasis_angle90_offset1 |  |  |
| 71 |  | GLCMEnergy_angle90_offset4 | ShortRunEmphasis_angle90_offset4 |  |  |
| 72 |  | GLCMEnergy_angle90_offset7 | ShortRunEmphasis_angle90_offset7 |  |  |
| 73 |  | GLCMEntropy_AllDirection_offset1 | RunLengthNonuniformity_AllDirection_offset1 |  |  |
| 74 |  | GLCMEntropy_AllDirection_offset1_SD | RunLengthNonuniformity_AllDirection_offset1_SD |  |  |
| 75 |  | GLCMEntropy_AllDirection_offset4 | RunLengthNonuniformity_AllDirection_offset4 |  |  |
| 76 |  | GLCMEntropy_AllDirection_offset4_SD | RunLengthNonuniformity_AllDirection_offset4_SD |  |  |
| 77 |  | GLCMEntropy_AllDirection_offset7 | RunLengthNonuniformity_AllDirection_offset7 |  |  |
| 78 |  | GLCMEntropy_AllDirection_offset7_SD | RunLengthNonuniformity_AllDirection_offset7_SD |  |  |
| 79 |  | GLCMEntropy_angle0_offset1 | RunLengthNonuniformity_angle0_offset1 |  |  |
| 80 |  | GLCMEntropy_angle0_offset4 | RunLengthNonuniformity_angle0_offset4 |  |  |
| 81 |  | GLCMEntropy_angle0_offset7 | RunLengthNonuniformity_angle0_offset7 |  |  |
| 82 |  | GLCMEntropy_angle135_offset1 | RunLengthNonuniformity_angle135_offset1 |  |  |
| 83 |  | GLCMEntropy_angle135_offset4 | RunLengthNonuniformity_angle135_offset4 |  |  |
| 84 |  | GLCMEntropy_angle135_offset7 | RunLengthNonuniformity_angle135_offset7 |  |  |
| 85 |  | GLCMEntropy_angle45_offset1 | RunLengthNonuniformity_angle45_offset1 |  |  |
| 86 |  | GLCMEntropy_angle45_offset4 | RunLengthNonuniformity_angle45_offset4 |  |  |
| 87 |  | GLCMEntropy_angle45_offset7 | RunLengthNonuniformity_angle45_offset7 |  |  |
| 88 |  | GLCMEntropy_angle90_offset1 | RunLengthNonuniformity_angle90_offset1 |  |  |
| 89 |  | GLCMEntropy_angle90_offset4 | RunLengthNonuniformity_angle90_offset4 |  |  |
| 90 |  | GLCMEntropy_angle90_offset7 | RunLengthNonuniformity_angle90_offset7 |  |  |
| 91 |  | HaralickCorrelation_AllDirection_offset1 | LongRunHighGreyLevelEmphasis_AllDirection_offset1 |  |  |
| 92 |  | HaralickCorrelation_AllDirection_offset1_SD | LongRunHighGreyLevelEmphasis_AllDirection_offset1_SD |  |  |
| 93 |  | HaralickCorrelation_AllDirection_offset4 | LongRunHighGreyLevelEmphasis_AllDirection_offset4 |  |  |
| 94 |  | HaralickCorrelation_AllDirection_offset4_SD | LongRunHighGreyLevelEmphasis_AllDirection_offset4_SD |  |  |
| 95 |  | HaralickCorrelation_AllDirection_offset7 | LongRunHighGreyLevelEmphasis_AllDirection_offset7 |  |  |
| 96 |  | HaralickCorrelation_AllDirection_offset7_SD | LongRunHighGreyLevelEmphasis_AllDirection_offset7_SD |  |  |
| 97 |  | HaralickCorrelation_angle0_offset1 | LongRunHighGreyLevelEmphasis_angle0_offset1 |  |  |
| 98 |  | HaralickCorrelation_angle0_offset4 | LongRunHighGreyLevelEmphasis_angle0_offset4 |  |  |
| 99 |  | HaralickCorrelation_angle0_offset7 | LongRunHighGreyLevelEmphasis_angle0_offset7 |  |  |
| 100 |  | HaralickCorrelation_angle135_offset1 | LongRunHighGreyLevelEmphasis_angle135_offset1 |  |  |
| 101 |  | HaralickCorrelation_angle135_offset4 | LongRunHighGreyLevelEmphasis_angle135_offset4 |  |  |
| 102 |  | HaralickCorrelation_angle135_offset7 | LongRunHighGreyLevelEmphasis_angle135_offset7 |  |  |
| 103 |  | HaralickCorrelation_angle45_offset1 | LongRunHighGreyLevelEmphasis_angle45_offset1 |  |  |
| 104 |  | HaralickCorrelation_angle45_offset4 | LongRunHighGreyLevelEmphasis_angle45_offset4 |  |  |
| 105 |  | HaralickCorrelation_angle45_offset7 | LongRunHighGreyLevelEmphasis_angle45_offset7 |  |  |
| 106 |  | HaralickCorrelation_angle90_offset1 | LongRunHighGreyLevelEmphasis_angle90_offset1 |  |  |
| 107 |  | HaralickCorrelation_angle90_offset4 | LongRunHighGreyLevelEmphasis_angle90_offset4 |  |  |
| 108 |  | HaralickCorrelation_angle90_offset7 | LongRunHighGreyLevelEmphasis_angle90_offset7 |  |  |
| 109 |  | Angular second moment | LongRunLowGreyLevelEmphasis_AllDirection_offset1 |  |  |
| 110 |  | Contrast | LongRunLowGreyLevelEmphasis_AllDirection_offset1_SD |  |  |
| 111 |  | Haralick entropy | LongRunLowGreyLevelEmphasis_AllDirection_offset4 |  |  |
| 112 |  | Haralick variance | LongRunLowGreyLevelEmphasis_AllDirection_offset4_SD |  |  |
| 113 |  | Sum average | LongRunLowGreyLevelEmphasis_AllDirection_offset7 |  |  |
| 114 |  | Sum entropy | LongRunLowGreyLevelEmphasis_AllDirection_offset7_SD |  |  |
| 115 |  | Sum variance | LongRunLowGreyLevelEmphasis_angle0_offset1 |  |  |
| 116 |  | Difference entropy | LongRunLowGreyLevelEmphasis_angle0_offset4 |  |  |
| 117 |  | Difference variance | LongRunLowGreyLevelEmphasis_angle0_offset7 |  |  |
| 118 |  | Inverse difference moment | LongRunLowGreyLevelEmphasis_angle135_offset1 |  |  |
| 119 |  | InverseDifferenceMoment_AllDirection_offset1 | LongRunLowGreyLevelEmphasis_angle135_offset4 |  |  |
| 120 |  | InverseDifferenceMoment_AllDirection_offset1_SD | LongRunLowGreyLevelEmphasis_angle135_offset7 |  |  |
| 121 |  | InverseDifferenceMoment_AllDirection_offset4 | LongRunLowGreyLevelEmphasis_angle45_offset1 |  |  |
| 122 |  | InverseDifferenceMoment_AllDirection_offset4_SD | LongRunLowGreyLevelEmphasis_angle45_offset4 |  |  |
| 123 |  | InverseDifferenceMoment_AllDirection_offset7 | LongRunLowGreyLevelEmphasis_angle45_offset7 |  |  |
| 124 |  | InverseDifferenceMoment_AllDirection_offset7_SD | LongRunLowGreyLevelEmphasis_angle90_offset1 |  |  |
| 125 |  | InverseDifferenceMoment_angle0_offset1 | LongRunLowGreyLevelEmphasis_angle90_offset4 |  |  |
| 126 |  | InverseDifferenceMoment_angle0_offset4 | LongRunLowGreyLevelEmphasis_angle90_offset7 |  |  |
| 127 |  | InverseDifferenceMoment_angle0_offset7 | ShortRunHighGreyLevelEmphasis_AllDirection_offset1 |  |  |
| 128 |  | InverseDifferenceMoment_angle135_offset1 | ShortRunHighGreyLevelEmphasis_AllDirection_offset1_SD |  |  |
| 129 |  | InverseDifferenceMoment_angle135_offset4 | ShortRunHighGreyLevelEmphasis_AllDirection_offset4 |  |  |
| 130 |  | InverseDifferenceMoment_angle135_offset7 | ShortRunHighGreyLevelEmphasis_AllDirection_offset4_SD |  |  |
| 131 |  | InverseDifferenceMoment_angle45_offset1 | ShortRunHighGreyLevelEmphasis_AllDirection_offset7 |  |  |
| 132 |  | InverseDifferenceMoment_angle45_offset4 | ShortRunHighGreyLevelEmphasis_AllDirection_offset7_SD |  |  |
| 133 |  | InverseDifferenceMoment_angle45_offset7 | ShortRunHighGreyLevelEmphasis_angle0_offset1 |  |  |
| 134 |  | InverseDifferenceMoment_angle90_offset1 | ShortRunHighGreyLevelEmphasis_angle0_offset4 |  |  |
| 135 |  | InverseDifferenceMoment_angle90_offset4 | ShortRunHighGreyLevelEmphasis_angle0_offset7 |  |  |
| 136 |  | InverseDifferenceMoment_angle90_offset7 | ShortRunHighGreyLevelEmphasis_angle135_offset1 |  |  |
| 137 |  | Inertia_AllDirection_offset1 | ShortRunHighGreyLevelEmphasis_angle135_offset4 |  |  |
| 138 |  | Inertia_AllDirection_offset1_SD | ShortRunHighGreyLevelEmphasis_angle135_offset7 |  |  |
| 139 |  | Inertia_AllDirection_offset4 | ShortRunHighGreyLevelEmphasis_angle45_offset1 |  |  |
| 140 |  | Inertia_AllDirection_offset4_SD | ShortRunHighGreyLevelEmphasis_angle45_offset4 |  |  |
| 141 |  | Inertia_AllDirection_offset7 | ShortRunHighGreyLevelEmphasis_angle45_offset7 |  |  |
| 142 |  | Inertia_AllDirection_offset7_SD | ShortRunHighGreyLevelEmphasis_angle90_offset1 |  |  |
| 143 |  | Inertia_angle0_offset1 | ShortRunHighGreyLevelEmphasis_angle90_offset4 |  |  |
| 144 |  | Inertia_angle0_offset4 | ShortRunHighGreyLevelEmphasis_angle90_offset7 |  |  |
| 145 |  | Inertia_angle0_offset7 | ShortRunLowGreyLevelEmphasis_AllDirection_offset1 |  |  |
| 146 |  | Inertia_angle135_offset1 | ShortRunLowGreyLevelEmphasis_AllDirection_offset1_SD |  |  |
| 147 |  | Inertia_angle135_offset4 | ShortRunLowGreyLevelEmphasis_AllDirection_offset4 |  |  |
| 148 |  | Inertia_angle135_offset7 | ShortRunLowGreyLevelEmphasis_AllDirection_offset4_SD |  |  |
| 149 |  | Inertia_angle45_offset1 | ShortRunLowGreyLevelEmphasis_AllDirection_offset7 |  |  |
| 150 |  | Inertia_angle45_offset4 | ShortRunLowGreyLevelEmphasis_AllDirection_offset7_SD |  |  |
| 151 |  | Inertia_angle45_offset7 | ShortRunLowGreyLevelEmphasis_angle0_offset1 |  |  |
| 152 |  | Inertia_angle90_offset1 | ShortRunLowGreyLevelEmphasis_angle0_offset4 |  |  |
| 153 |  | Inertia_angle90_offset4 | ShortRunLowGreyLevelEmphasis_angle0_offset7 |  |  |
| 154 |  | Inertia_angle90_offset7 | ShortRunLowGreyLevelEmphasis_angle135_offset1 |  |  |
| 155 |  |  | ShortRunLowGreyLevelEmphasis_angle135_offset4 |  |  |
| 156 |  |  | ShortRunLowGreyLevelEmphasis_angle135_offset7 |  |  |
| 157 |  |  | ShortRunLowGreyLevelEmphasis_angle45_offset1 |  |  |
| 158 |  |  | ShortRunLowGreyLevelEmphasis_angle45_offset4 |  |  |
| 159 |  |  | ShortRunLowGreyLevelEmphasis_angle45_offset7 |  |  |
| 160 |  |  | ShortRunLowGreyLevelEmphasis_angle90_offset1 |  |  |
| 161 |  |  | ShortRunLowGreyLevelEmphasis_angle90_offset4 |  |  |
| 162 |  |  | ShortRunLowGreyLevelEmphasis_angle90_offset7 |  |  |

Abbreviations: GLCM = gray level co-occurrence matrix; GLRML = gray level run length matrix; GLSZM = gray level size zone matrix.
