# Supplementary material for: Radiomic Analysis of Contrast-Enhanced Mammography With Different Image Types: Classification of Breast Lesions
Source: Front Oncol. 2021 May 28;11:600546. doi: 10.3389/fonc.2021.600546 (PMC8195270; doi:10.3389/fonc.2021.600546)
Supplement: Supplementary file 2 [file Data_Sheet_1.docx]

**Supplemental Figure**

**
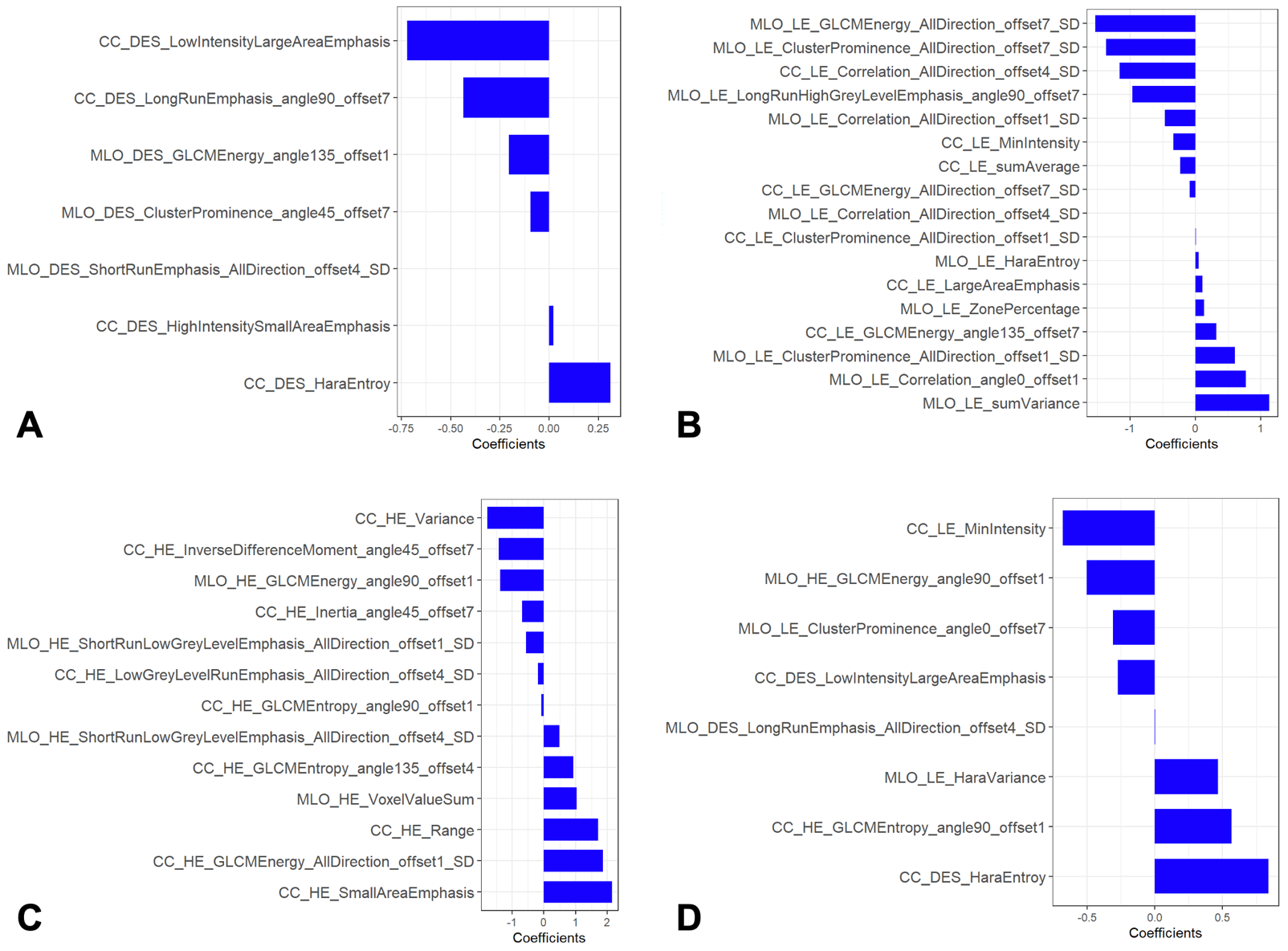
**

Supplemental Figure 1. The selected radiomic features for constructing radiomics models.

The figure shows the selected radiomic features and their corresponding coefficients (blue bars) for constructing the classification models by using (A) dual-energy subtraction (DES), (B) low-energy (LE), (C) high-energy (HE) images, and (D) all of the three types of contrast-enhanced mammography (CEM) images.

CC = craniocaudal; MLO = mediolateral oblique; SD = standard deviation.

**
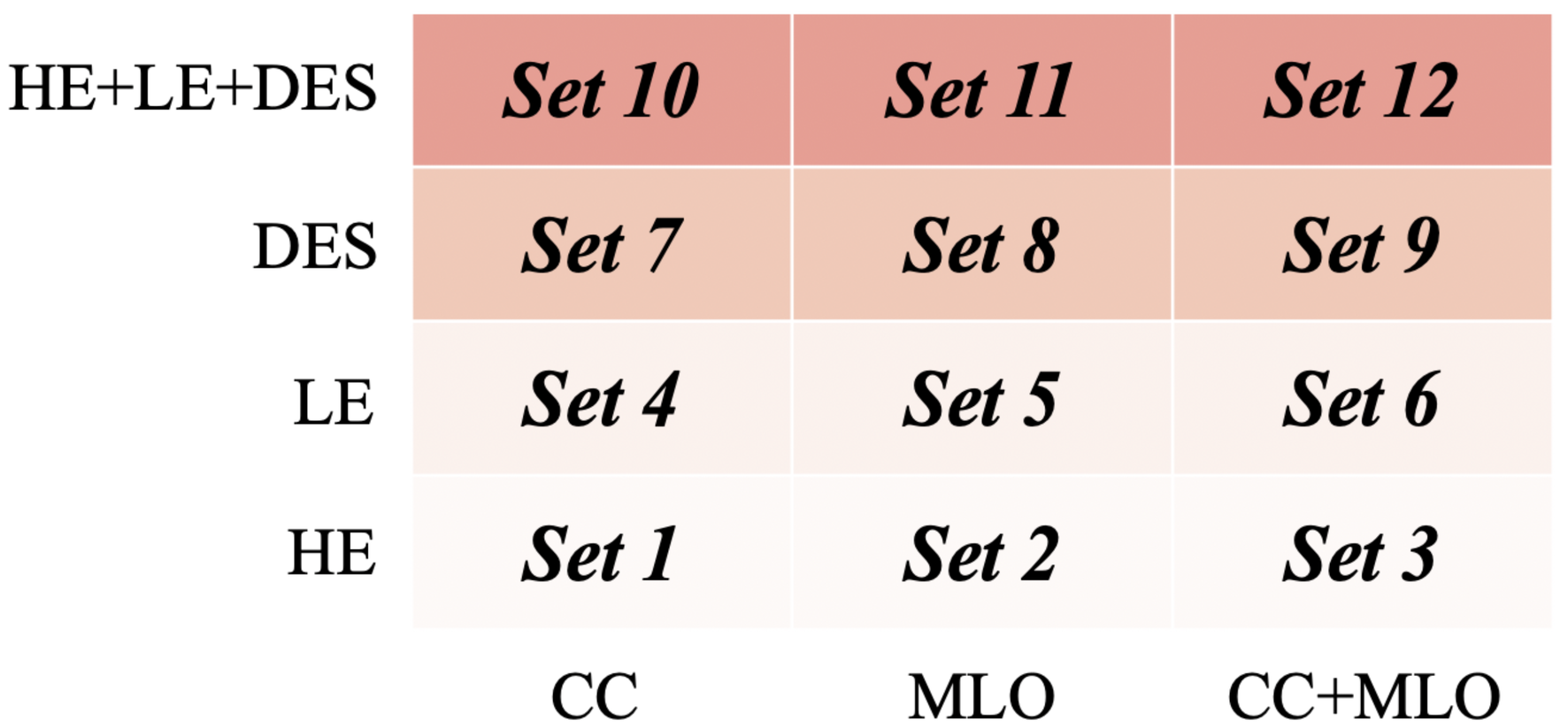
**

Supplemental Figure 2. Feature datasets.

For each lesion, a total of 6 region-of-interests (ROIs) were delineated, thus producing 6 original feature datasets, including HE-CC, HE-MLO, LE-CC, LE-MLO, DES-CC and DES-MLO datasets (Datasets 1, 2, 4,5, 7 and 8). We combined the original 6 datasets to generate 6 other datasets for each lesion (Datasets 3, 6, 9, 10, 11 and 12).

HE = high-energy; LE = low-energy; DES = dual-energy subtraction; CC = craniocaudal; MLO = mediolateral oblique.


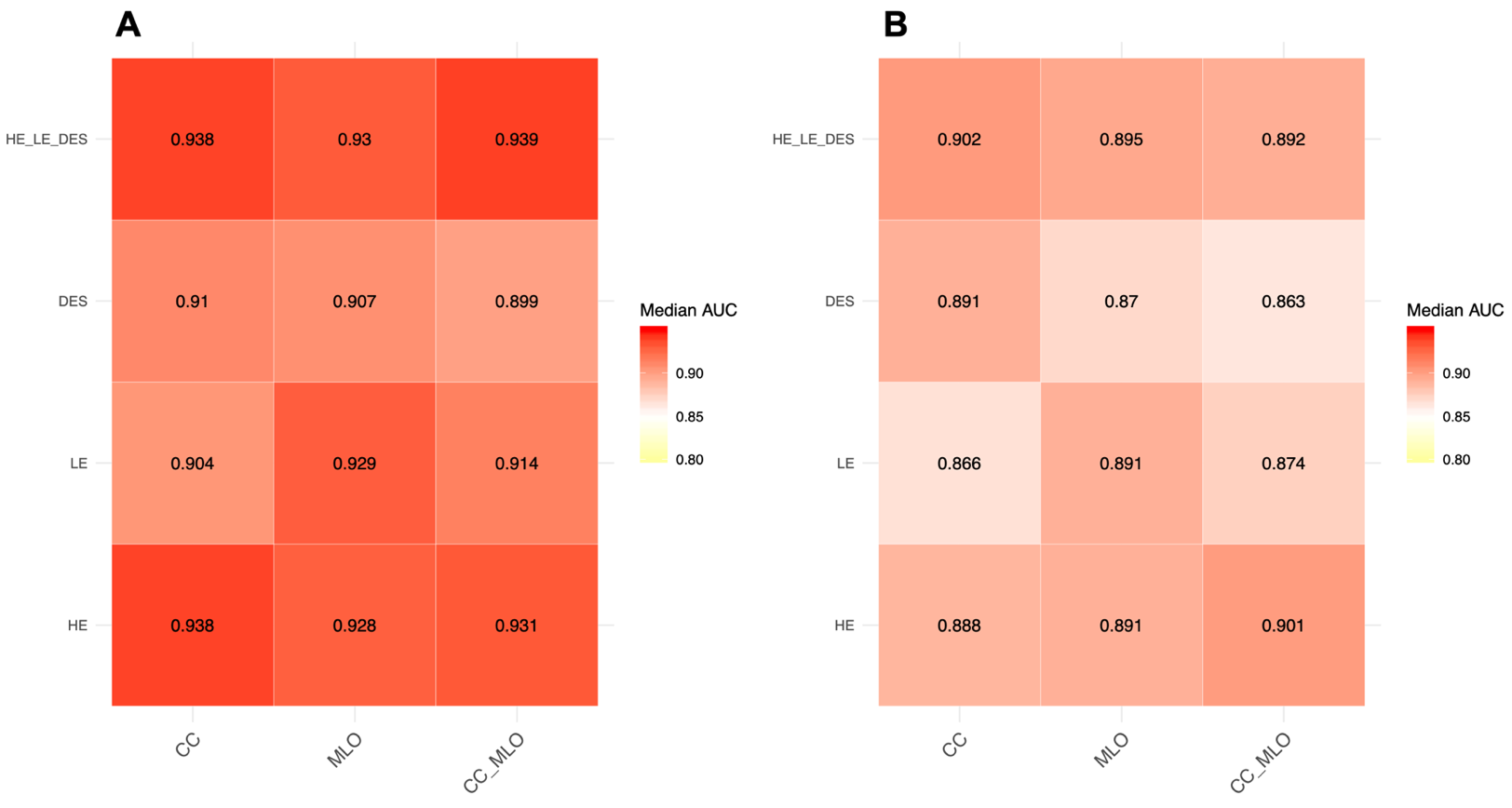


Supplemental Figure 3. The heatmaps of the median AUC values of different datasets.

The median AUCs of all the models constructed with the 12 feature datasets (Supplemental Figure 2) are basically in parallel with the mean AUCs of the corresponding models (Table 3). The models constructed with HE images or a combination of HE, LE and DES images achieved the highest median AUCs (median AUCs = 0.901 and 0.892, respectively).

(A) Training and (B) testing sets. HE = high-energy; LE = low-energy; DES = dual-energy subtraction; AUC = area under the receiver operating characteristic curve; CC= craniocaudal; MLO = mediolateral oblique.
